# Improving Time to Antibiotic Administration in Open Fractures

**DOI:** 10.1097/pq9.0000000000000890

**Published:** 2026-07-28

**Authors:** Fiona A. Fimmel, Emily M. Rheaume, Kelly S. Falcone, Vincent R. Alexander, Sean Bartlett, Laurie H. Johnson, Adam A. Vukovic

**Affiliations:** From the * Department of Pediatrics, University of Cincinnati College of Medicine, Cincinnati, Ohio; † Pediatric General and Thoracic Surgery, Cincinnati Children’s Hospital Medical Center, Cincinnati, Ohio; ‡ Pediatric Orthopedic Surgery Department, Cincinnati Children’s Hospital Medical Center, Cincinnati, Ohio; § Division of Emergency Medicine, Cincinnati Children’s Hospital Medical Center, Cincinnati, Ohio; ¶ Emergency Department Patient Services, Cincinnati Children’s Hospital Medical Center, Cincinnati, Ohio.

## Abstract

**Introduction:**

Timely antibiotic treatment for pediatric open long-bone fractures is associated with a reduced risk of infection. National guidelines recommend administering intravenous antibiotics within 60 minutes of presentation to the emergency department (ED). Historically, this study’s level 1 pediatric trauma center has not met this benchmark. The objective of this study was to improve antibiotic administration time for pediatric patients with open long-bone fractures from a baseline of 113 to 60 minutes or less.

**Methods:**

A multidisciplinary team used serial Plan-Do-Study-Act cycles to implement and refine ED processes. Key interventions included targeted education for stakeholders, an updated order set with standardized antibiotic recommendations, modification of the existing orthopedic triage workflow, and quality review of eligible cases with feedback provided to treatment teams. Data were analyzed using X-moving range control charts to identify signals of change. The primary outcome measure was the time to appropriate antibiotic administration after arrival at the ED. Use of the newly introduced order set was tracked as a process measure, and time to analgesic and fracture reduction were balancing measures.

**Results:**

Data collected from September 2020 to March 2025 included 111 patients, 60 of whom were seen postintervention (starting in April 2023). Time to antibiotic administration improved from 113 to 36 minutes. There was a modest improvement in order set usage. Balancing measure times were not negatively impacted.

**Conclusions:**

The use of an iterative multimodal quality improvement package reduced the time to antibiotic administration and improved adherence to evidence-based protocols for the initial treatment of pediatric open long-bone fractures.

## INTRODUCTION

### Problem Description

Open fractures involving skin disruption and subsequent bone exposure have higher rates of infection and soft tissue complications than closed fractures and are associated with poorer outcomes.^1^ Prompt administration of intravenous (IV) antibiotics—ideally within 1 hour of patient presentation—is an important factor in decreasing the rate of infections in these injuries.^2,3^ Historically, this study’s pediatric emergency department (ED) has not met this benchmark, prompting exploration into how timely antibiotic administration could be reliably achieved.

### Available Knowledge

Open long-bone fractures are characterized via Gustilo-Anderson grading, which assigns grades I–III (IIIA–C) based on skin laceration length, soft tissue damage, degree of bone coverage, fracture comminution, and vascular disruption.^4^ Existing research, specifically in adult orthopedic and trauma medicine, recommends that IV antibiotics for open fractures cover Gram-positive skin flora, Gram-negative organisms for higher grade fractures, and in the case of fecal or clostridial contamination, should include high-dose penicillin.^5^ Current recommendations for open fractures encourage operative irrigation and debridement within 24 hours of injury.^3^ Prompt administration of appropriate IV antibiotics while awaiting definitive care is an important factor in preventing infection and optimizing healing, and greater delay between patient presentation to the ED and antibiotic administration is associated with higher rates of infection.^6,7^

### Rationale

Fractures are a common pediatric injury treated in the ED, and although open fractures are less common than closed fractures, they warrant treatment considerations that staff may have limited experience managing.^8^ Existing workflows and standardized practices in the study ED had not been optimized to facilitate prompt antibiotic administration in patients with open fractures. Although an order set for fracture management already existed, it did not provide guidance on antibiotic selection in accordance with EAST (Eastern Association for the Surgery of Trauma) Practice Management guidelines, creating a risk of practice variation and inappropriate antibiotic administration. Quality improvement (QI) initiatives, including provider education and standardized order sets, have previously been used to reduce variation in antibiotic timing and improve this metric in both pediatric and adult EDs; prior studies similar to this have been performed in different patient populations (eg, adults) or with different target times to antibiotic administration despite EAST Practice Management guidelines.^9–11^ Benchmarking efforts that use multiple strategies (including provider feedback and targeted QI plans) have been associated with significantly improved outcome measures in healthcare settings.^12^

### Specific Aims

The SMART (Specific, Measurable, Actionable, Relevant, Timely) aim of this study was to reduce the time to IV antibiotic administration for pediatric patients with open long-bone fractures from a baseline of 113 to 60 minutes or less during a 16-month study period.

## METHODS

### Context

**Fig. 1.**
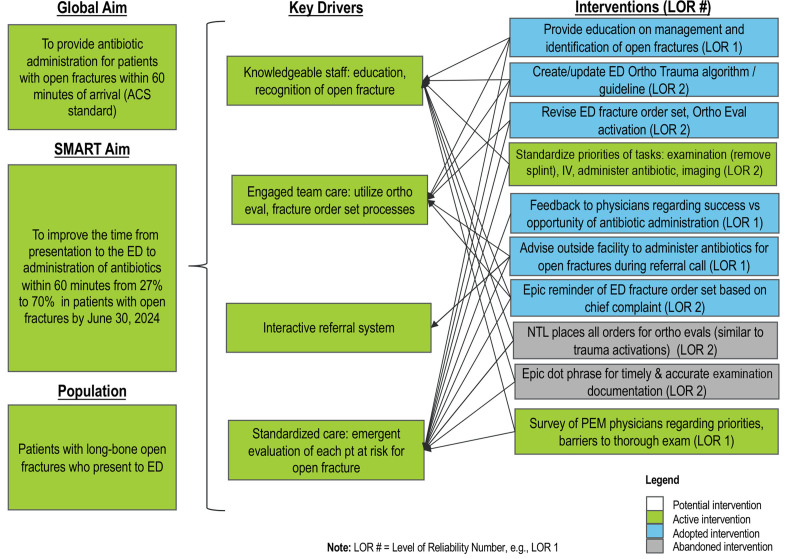
Key driver diagram. ACS, American College of Surgeons; LOR #, level of reliability number; NTL, nursing team lead; Ortho Eval, orthopedic evaluation; PEM, pediatric emergency medicine; SMART, Specific, Measurable, Actionable, Relevant, Timely. This work is licensed under Creative Commons Attribution Non-Commercial Sharealike 4.0 International.

The study ED is a freestanding level 1 trauma center that sees more than 60,000 patients per year. It is housed within an academic hospital that employs more than 200 pediatric residents who rotate through the department, as well as emergency and family medicine resident rotators and medical students. In addition to the resident physicians, the ED treatment team includes pediatric emergency medicine faculty and fellows, pediatricians, advanced practitioners, registered nurses, respiratory therapists, paramedics, child life specialists, and patient care assistants. Patients who present to the ED with orthopedic injuries are initially triaged by ED registered nurses using the Emergency Severity Index tool to determine injury severities and evaluation timelines; patients will either return to the lobby, be immediately roomed and activated as an “orthopedic evaluation”—where a specific team of providers is called to do an immediate in-room assessment—or be evaluated in the shock trauma suite. (**See figure 1, Supplemental Digital Content 1**, which displays the orthopedic evaluation flowsheet, https://links.lww.com/PQ9/A767.)^13,14^

### Interventions

The multidisciplinary study team consisted of pediatric emergency medicine physicians and fellows, trauma and emergency medicine nurses, and a resident physician. The team theorized drivers that likely impacted timely access to antibiotics in open long-bone fractures and created a key driver diagram that identified potential opportunities for intervention (Fig. 1). Key drivers included knowledgeable and engaged stakeholders, an interactive ED referral system, optimized workflows for orthopedic evaluation and medication ordering, and care standardization.

Multiple interventions were considered during this improvement initiative. The primary interventions adopted during this study included an optimized preexisting ED fracture order set, a refined and standardized evaluation process for fractures, and ongoing education and feedback to members of the care team. These interventions were refined through serial Plan-Do-Study-Act cycles, with modifications informed by iterative case reviews.

The team worked with the hospital’s electronic medical record (EMR) specialist to add first-line antibiotics with appropriate dosing to the preexisting order set, including hyperlinks to guide additional coverage as needed. Fracture pattern descriptions were provided, as were alternative therapies for patients allergic to first-line treatments. The recommended antibiotics included cefazolin for Gustilo type I and II fractures, ceftriaxone and metronidazole for Gustilo type III, and piperacillin-tazobactam for fecal or water contamination, with additional recommendations for patients with penicillin allergies.

Education regarding patients with potential open long-bone fractures included both general and specific measures. Signage with information about fracture guidelines and the updated order set was prominently displayed in provider-facing areas of the ED. Tailored study materials were also incorporated into routine education for nurses and resident physicians during ED rotations. Information about the study goals and ongoing clinical metrics was communicated in nursing and provider team meetings. Open fracture cases were regularly discussed by the study team. Nurses and providers who cared for incident patients were emailed to solicit feedback and, if warranted, provide education.

Standard orthopedic and trauma evaluations were updated to include specific prompts to inquire about bleeding at the injury site and evaluate skin breakage in fracture patients based on the chief complaint. Triage guidelines developed to facilitate the prompt evaluation of orthopedic trauma were expanded to include assessment of open fractures (**Supplemental Digital Content 1**, https://links.lww.com/PQ9/A767). The ED referral center was also encouraged to inquire about the potential for open fractures when patients were referred from outside hospitals and to recommend antibiotic administration before transfer.

### Study of Interventions

A data extraction specialist used the hospital EMR to identify eligible patients and obtain relevant data points. They extracted data from the EMR and stored it in a password-protected computer and file accessible only to a limited number of study representatives (ER). Data included patient demographic information and timestamps for study-relevant events, such as ED arrival time, antibiotic order and administration time, and time to sedation for fracture reduction. Cases where antibiotics were administered before ED arrival were excluded. Control charts were constructed and tracked throughout the study to monitor intervention success and detect special-cause variation. Interventions were plotted on the same chart to understand their associations and impacts.

### Measures

The primary outcome measure was the time to antibiotic administration. This was calculated as the time antibiotics were started minus the time of patient arrival to the ED, measured in minutes; these data points were reliable data points extractable from the EMR. As a process measure, the percentage of encounters of patients with open fractures in which antibiotics were ordered using the order set (both before and after modification) was tracked. This was calculated in increments of 5 patient encounters, with the numerator being patient encounters in which the order set was used and the denominator being all encounters within that set of 5. Acknowledging the competing priorities related to long-bone fractures, time to analgesic administration and time to procedural sedation for closed fracture reduction were tracked as balancing measures. Time to closed fracture reduction was defined as the time from the first dose of sedative medication administered for orthopedic reduction to the patient’s ED arrival, expressed in minutes. Time to analgesic administration was calculated in the same manner, but with reference to the first dose of analgesic medication.

### Analysis

All measures were tracked on appropriate statistical process control charts. Standard rules for interpreting control charts were used to identify special-cause variation in the study data; specifically, a shift was defined as 8 or more points above or below the centerline, with a theory explaining its cause.^15^ For the outcome measure, this study used an X-MR (moving range) chart, in which each data point represents the time from arrival to antibiotic administration for an individual patient encounter. For the process measure, each data point on the P-chart represents the percentage of eligible patients whose antibiotics were ordered via the order set, calculated per group of 5 patients. The study team tracked balancing measures using an X-MR chart, with each data point representing the time from an individual patient’s arrival to the start of sedation for fracture reduction or the time of analgesia administration. For each chart, the centerline represents the mean, and the control limits represent 3 SDs above or below the mean.

### Ethical Considerations

This study does not pose any unique ethical considerations. Before the onset of this work, divisional research and QI leadership reviewed this study and determined it to be QI and thus not subject to a full institutional review board review.

## RESULTS

Data from September 2020 through March 2025 were collected, including 111 patients. The baseline period included 51 patients, and the improvement period included 60. Patients’ ages ranged from 3 to 17 years, and they were evaluated for fractures of the humerus, radius/ulna, femur, tibia/fibula, and olecranon (Table 1).

**Table 1.** Characteristics of Study Patients

Race, n (%)	
White	85 (76)
Black of African American	18 (16)
Not listed	5 (4)
White, American Indian, and Alaska Native	2 (2)
White, Asian	1 (1)
Ethnicity, n (%)	
Non-Hispanic	102 (91)
Hispanic	6 (5)
Unknown	3 (3)
Sex, n (%)	
Male	77 (69)
Female	34 (30)
Age, y, n (%)	
0–5	11 (10)
6–12	67 (60)
12 and older	33 (29)
Bone involved, n (%)	
Radius and/or ulna	60 (54)
Tibia and/or fibula	38 (34)
Humerus	6 (5)
Femur	6 (5)
Olecranon	1 (1)
Antibiotics used before study, n (%)	
Cefazolin	46 (90)
Clindamycin	2 (4)
Ampicillin-sulbactam	2 (4)
Unrecorded	1 (2)
Antibiotics used after study, n (%)	
Cefazolin	57 (95)
Clindamycin	1 (2)
Ceftriaxone	1 (2)
Piperacillin-tazobactam	1 (2)

**Fig. 2.**
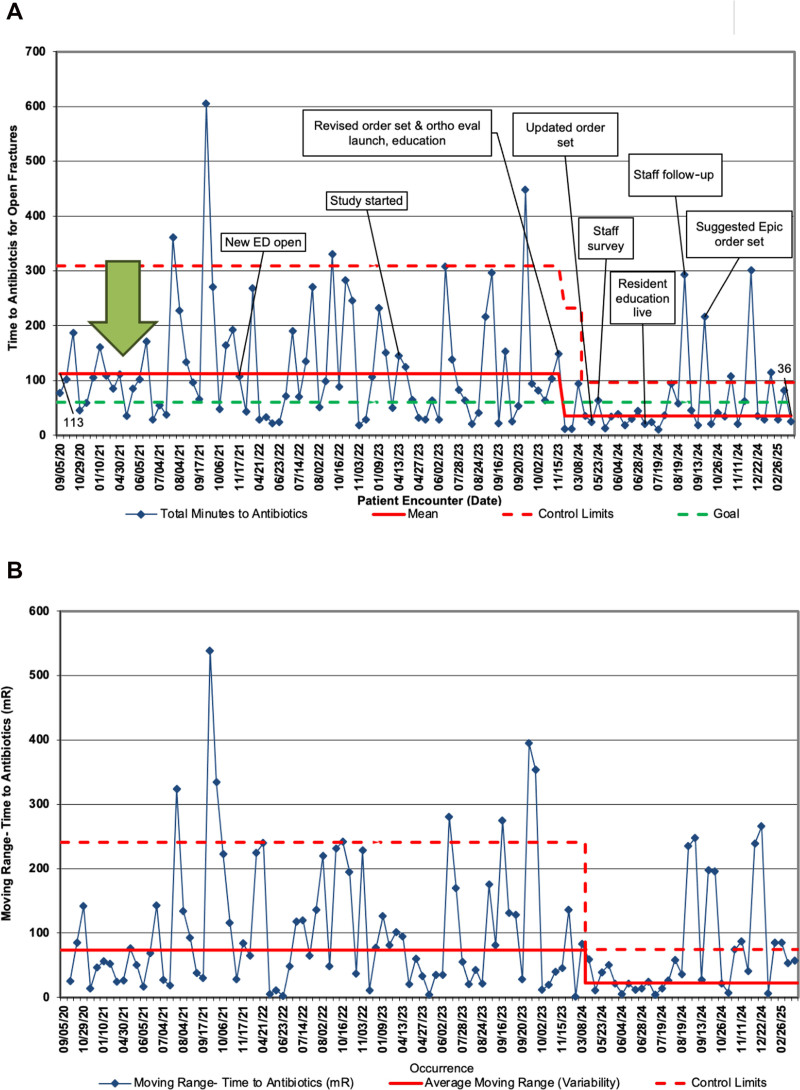
Time to antibiotic administration for open fractures (X-chart). Each data point represents a study patient. The solid red line indicates the mean. The interrupted red line indicates the upper control limit. The interrupted green line with the accompanying green arrow represents the study goal of 60 minutes. A, X-chart. B, MR (moving range) chart.

The study team implemented the revised order set in the EMR in November 2023, after which the average time to antibiotic administration for open fractures improved from a baseline of 113 to 36 minutes (Fig. 2). Following this centerline shift, several special-cause variation data points of concern were noted to exceed the upper control limit. Upon review of these events, 3 of the 5 involved patients presented with prearrival splints that limited visualization of the injury. Other factors contributing to delays in antibiotic administration included care disruption due to shift changes and missed education by a float provider. The moving range chart demonstrates reduced variation between successive patients, with a shift toward improvement in March 2024 from an average patient-to-patient difference of 74 to 23 minutes. Special-cause variation is associated with the patient encounters described earlier, in which prearrival splints were not removed.

Before this QI initiative, cefazolin was selected for management in 90% of cases; for those in whom cefazolin was not selected, 2 patients had documented allergies to β-lactams, 1 patient had extensive soft tissue damage and thus was administered ampicillin-sulbactam (although the study algorithm would have suggested piperacillin/tazobactam), and 1 patient received ampicillin-sulbactam in our ED after having been given cefazolin en route (though no justification was present in the provider’s documentation). After implementation, 95% of patients received cefazolin. Because information on the grade of each fracture was infrequently documented, the appropriateness of antibiotic choice was sometimes difficult to assess.

**Fig. 3.**
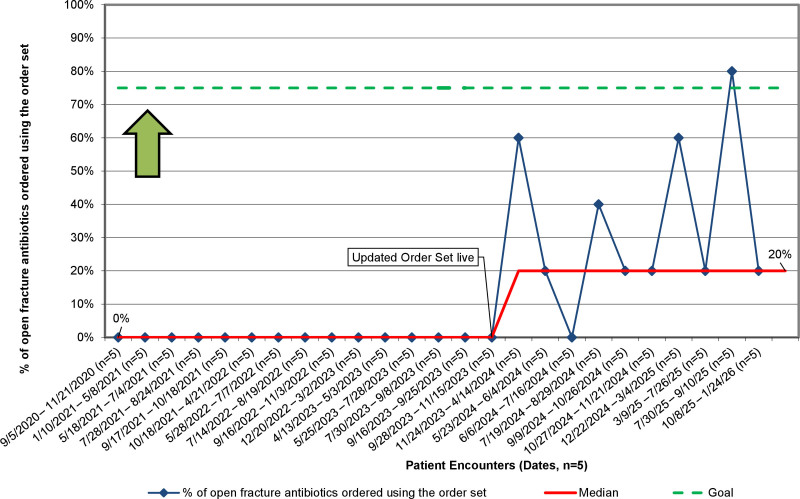
Percent of open fracture antibiotics ordered using the order set (P-chart). The solid red line indicates the mean. The interrupted green line with the accompanying green arrow represents the study goal of 75%.

**Fig. 4.**
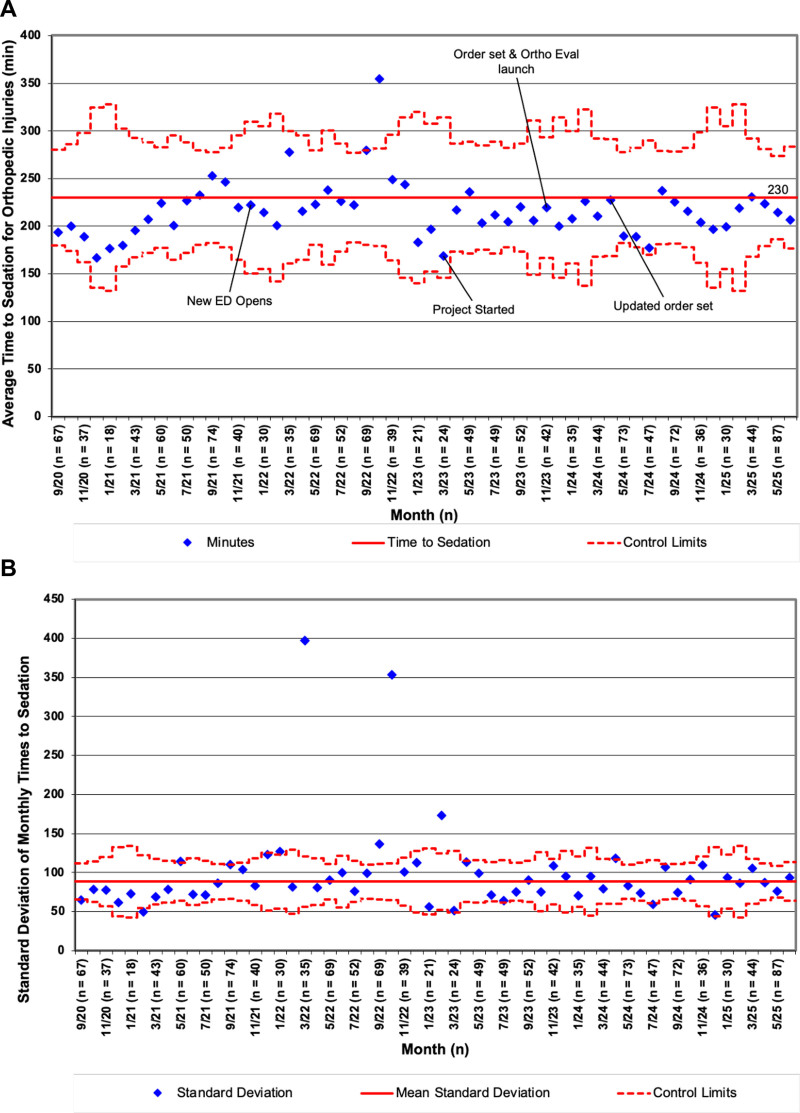
Time to sedation for closed fractures (X-chart). Ortho eval, orthopedic evaluation. Each data point represents the time to sedation for 1 patient undergoing closed fracture reduction. The solid red line indicates the mean. The interrupted red line indicates the upper and lower control limits. A, X-chart. B, S (SD) chart.

**Fig. 5.**
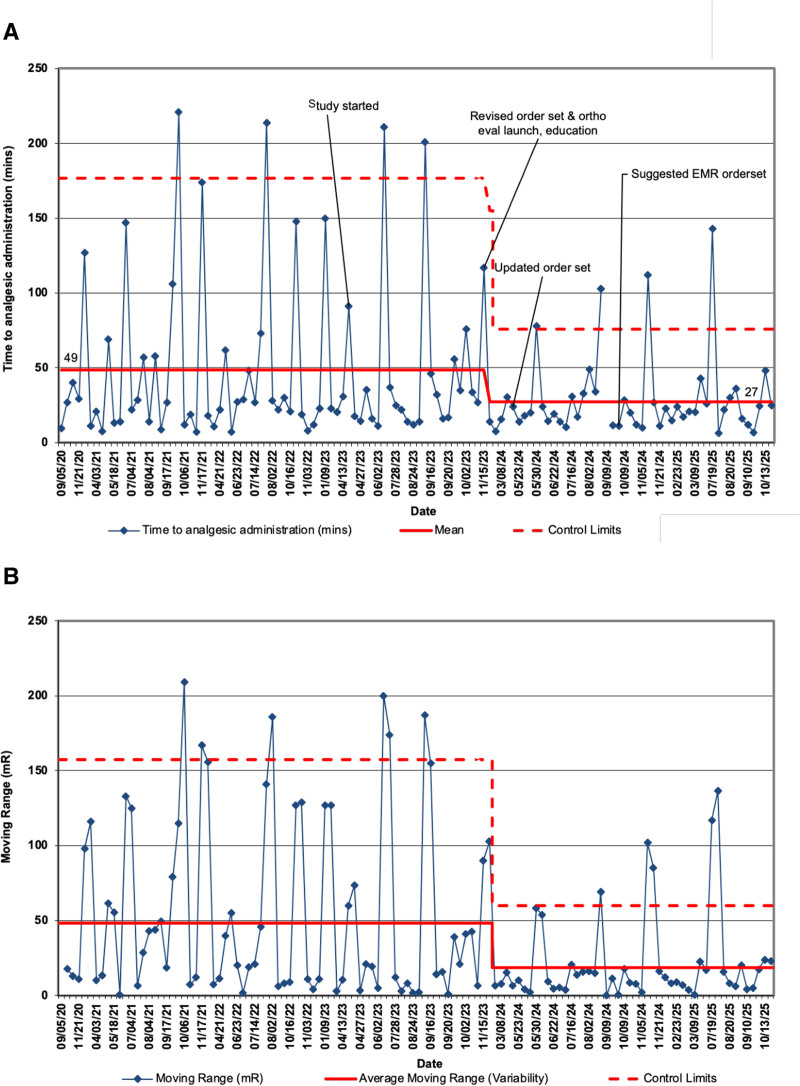
Time to analgesia administration (X-chart). A, X-chart. B, MR (moving range) chart.

Order set usage during relevant clinical encounters increased from 0% to 34% after implementation and provider and staff education. This centerline change includes 9 data points above the previous centerline with 1 data point on the centerline, which counts neither toward nor against accepted guidelines for a shift. These data include 3 data points beyond the conclusion of the original study period (Fig. 3). Throughout the study, the time to procedural sedation for fracture reduction remained unchanged at 210 minutes (Fig. 4), whereas the time to analgesia administration decreased slightly (Fig. 5).

## DISCUSSION

### Summary

Using QI methodology, this study improved the time to IV antibiotic administration for long-bone open fractures in our pediatric ED, reducing it from a baseline of 113 to 36 minutes, aligning with national guidelines.

### Interpretation

This improvement was appreciated following the introduction of the revised order set, new orthopedic evaluation guidelines, and a provider education program, and remained stable through several subsequent modifications and additions to primary interventions. Outcomes were accompanied by increased use of an existing fracture order set after modifications, underscoring the utility of standardized order sets in the ED. This process did not delay procedural sedation for fracture reduction or time to analgesic administration for open fractures.

This outcome is consistent with other QI studies targeting antibiotic administration for open fractures or other infectious processes, which aim to improve adherence to national guidelines.^4^ Open long-bone fractures are relatively uncommon injuries, even in a large pediatric ED, which can pose a challenge for providers to remain vigilant toward adhering to national guidelines. Standardizing orthopedic evaluations and antibiotic ordering, and providing education and real-time feedback, can result in appropriate, effective guidance for the care of these injuries.

Our multimodal QI package included successive rounds of provider education targeted at different provider groups in the ED (eg, nurses, attendings, or medical residents) using fliers, structured education, and individual feedback for providers involved in open fracture cases. The revised order set also provided guidance by providing links to open fracture-specific information as well as prompting the selection of appropriate antibiotics within 60 minutes or less. Before this study, a fracture-specific order set already existed, but it was rarely used for open or closed fractures. After modification and education, 31% of antibiotics ordered for open fractures were ordered using the modified order set. This improvement is in line with those observed in other studies but is still below the study goal of 70%.^10,11^ Suboptimal order set usage is due in part to individual ordering practices; although physicians often use order sets, many patients presenting with open fractures are initially seen by a multidisciplinary team in the trauma bay, where nursing representatives, who often do not use order sets, are responsible for order entry.

Although overall order set usage has only moderately increased, the attention to timely antibiotic ordering, including not only specific education but also case evaluation and follow-up interviews, has been disseminated among providers and resulted in a departmental culture change, as evidenced by sustained improvement and feedback from providers in postcase debriefs with the study team. In addition, the antibiotic choice guidelines provided by the order set remove human factors that might lead to an inappropriate agent being chosen for an open fracture. Interventions targeting the individual practice habits of ordering providers are an ongoing opportunity for future improvement.

Following implementation, cases of special-cause variation, where the time to antibiotic administration fell outside of expected system performance, were discussed to identify opportunities to improve timely administration. The most common reason for failed timely administration of antibiotics was not identifying skin breaks associated with fractures that were splinted before arrival at the study hospital. The study team used this observation to inform updates to the orthopedic injury workflow (Fig. 1), including the removal of splints applied at outlying hospitals. Ongoing work will focus on prompting residents and fellows to remove splints applied at outlying hospitals when evaluating a patient with a fractured extremity upon arrival at our institution. Competing interests, including varying provider comfort with splint removal, are barriers to this work and present opportunities for future improvement.

### Limitations

This study was limited by the relatively infrequent occurrence of open fractures presenting to the ED, which made timely evaluation of the impact of interventions challenging. The Gustilo-Anderson fracture grade was not recorded or extracted, making it challenging to assess the appropriateness of antibiotic choice. The generalizability of this study may be limited by the characteristics of the study hospital; for example, a large academic center with access to resources such as shock trauma suites with well-established trauma evaluation pathways, in-house orthopedists, and an EMR with dedicated EMR specialists. Certain features of the study intervention, such as provider education and order set standardization, could reasonably be implemented at facilities without these additional resources. Although both the COVID pandemic and the transition to a new ED space might have impacted the outcome measure, time to antibiotic administration remained consistent for more than 2 years after the ED transition and improved only after intentional improvement work on this outcome. A contemporaneous QI study aimed at improving time to analgesia administration in all fractures may have extraneously affected the balancing measure of time to analgesia administration. The incidence of wound infections and complications was not tracked, which would have represented the ultimate patient-centered outcome for our study measure. Future work could explore whether a shorter time to antibiotic administration could result in improved outcomes, including a lower risk of osteomyelitis.

## CONCLUDING SUMMARY

This study demonstrates that using QI methodology can improve the timely administration of IV antibiotics to patients with open long-bone fractures in a pediatric ED. Ongoing work should focus on sustainability and identifying and addressing factors that lead to variation.

## Supplementary Material
