# Supplementary figures and images for: Improving Time to Antibiotic Administration in Open Fractures
Source: Pediatr Qual Saf. 2026 Jul 28;11(4):e890. doi: 10.1097/pq9.0000000000000890 (PMC13412662; doi:10.1097/pq9.0000000000000890)

## Ortho Evaluation Process

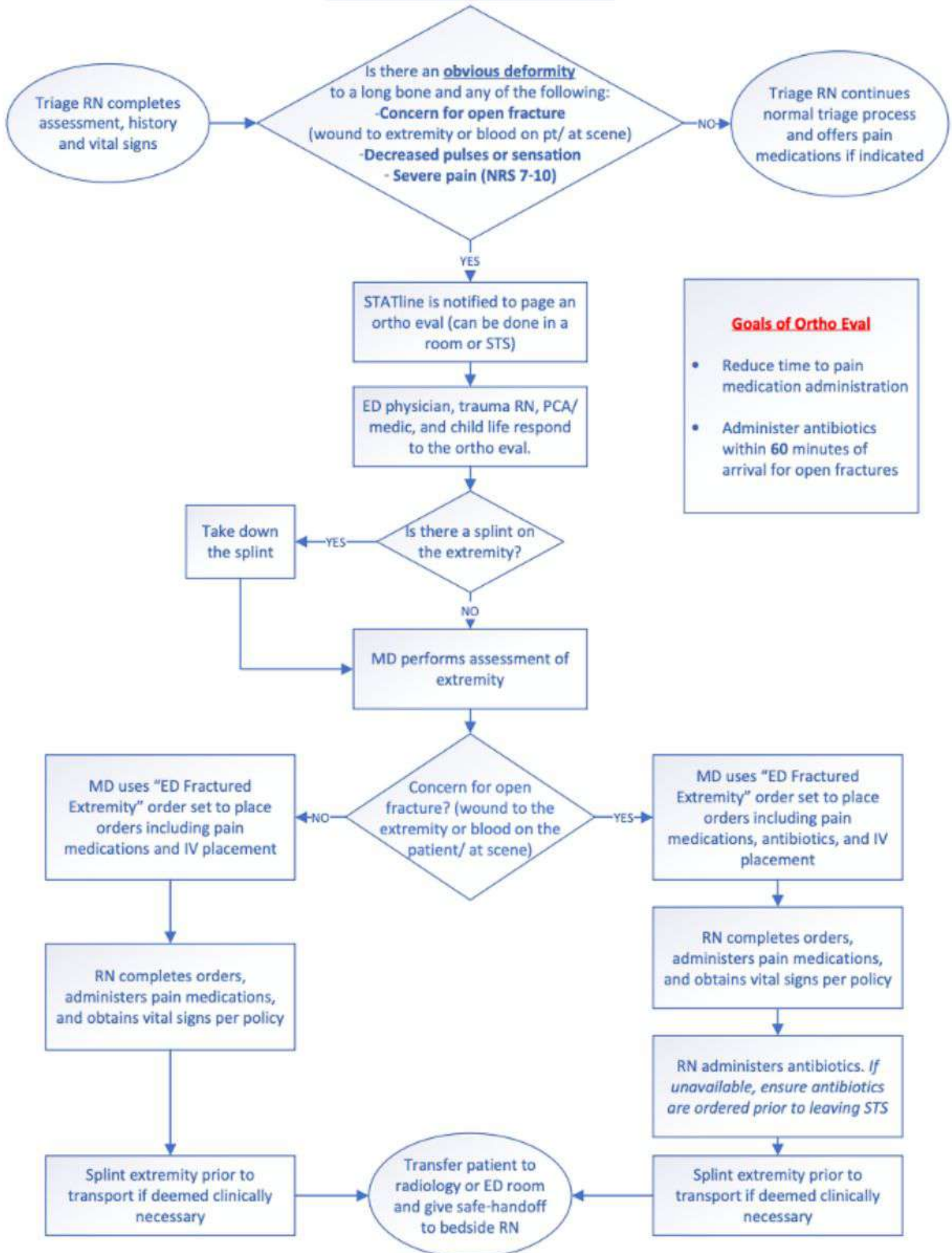

Supplement: Supplementary file 1 [file pqs-11-e890-s001.pdf]
